# Exploring the Impact of Remoteness and Socio-Economic Status on Child and Adolescent Injury-Related Mortality in Australia

**DOI:** 10.3390/children8010005

**Published:** 2020-12-24

**Authors:** Amy E. Peden, Richard C. Franklin

**Affiliations:** 1 School of Population Health, UNSW Sydney, Kensington, NSW 2052, Australia; 2 College of Public Health, Medical and Veterinary Sciences, James Cook University, Townsville, QLD 4811, Australia; richard.franklin@jcu.edu.au

**Keywords:** injury, child, adolescent, risk factor, rurality, socio-economic, determinants of health, road traffic injury, falls, poisoning, drowning, violence, self-harm, prevention, intervention, epidemiology

## Abstract

Injuries are a leading cause of harm for children. This study explores the impact of determinants of health on children (0–19 years) injury-related mortality (namely remoteness and socio-economic disadvantage, calculated using the index of relative socio-economic advantage and disadvantage (IRSAD)). Cause of death data from the Australian Bureau of Statistics were sourced for children in Australia between 1 July 2007 to 30 June 2017. Fifteen injury categories (ICD-10-AM external cause codes) were used. Burden and trends by injury mechanism were explored. A total of 5153 children died; with road traffic incidents (3.39 per 100,000 population), intentional self-harm (2.46) and drowning (0.72) being the leading mechanisms. Female fatality rates in very remote areas (8.73) were nine times higher than in major cities (Relative Risk [RR] = 8.73; 95% Confidence Interval [95% CI]: 4.23–18.00). Fatality rates increased with remoteness; very remote areas recording an injury-related fatality rated six times (RR = 5.84; 95% CI: 3.76–9.12) that of major city residents. Accidental poisoning and intentional self-harm fatalities were more likely in high IRSAD areas, while road traffic fatalities were more likely in low and mid socio-economic areas (X^2^ = 69.1; *p* < 0.001). People residing in regional and remote areas and from low socio-economic backgrounds already face significant health and lifestyle challenges associated with disadvantage. It is time to invest in injury prevention interventions for these populations, as well as upstream policy strategies to minimize any further preventable loss of life.

## 1. Introduction

Globally, injuries are a leading cause of mortality and morbidity for children and adolescents. In 2017, the Global Burden of Disease study estimated 4.48 million injury deaths globally, an increase of 5.3% since 1990 [1]. However, some progress is being made in reducing injury-related deaths, with both years of life lost and age-standardized mortality rates decreasing between 1990 and 2017 [1]. Injury-related morbidity is also a significant global concern. New cases of non-fatal injury are increasing, with 520 million cases recorded globally in 2017, while years lived with a disability also increased [1].

Understanding and preventing injuries is complex as they may be intentional or unintentional, be due to a range of mechanisms such as road transport, falls, drowning, burns, poisoning, interpersonal violence and suicide, impact all ages and require a range of strategies to prevent them from occurring [1,2,3,4]. As the risk factors and prevention strategies needed to address unintentional and intentional injuries often differ, there is a need for studies which explore the causes of injury by age groups.

Injury risk is impacted by external factors including determinants of health. Determinants of health, referred to as the causes of the causes [5], are the conditions in which people are born, grow, live, work and play which impact health [6]. Determinants which impact health, including injury risk, include socio-economic conditions, daily living conditions, education levels and individual health-related factors [7,8]. Addressing the determinants of health helps to prevent events from occurring [5]. Strategies to prevent injuries need to be designed to address injury risk using a range of strategies from downstream (individual level) to upstream (system level), otherwise they are likely to be ineffective, especially when used in isolation, and must address underlying determinants of health [9].

Geographical remoteness and socio-economic status are determinants which impact health, including injury [10,11]. Poorer health outcomes are seen in rural dwelling populations, with greater hospitalization rates and disease burden [12,13]. This increased injury risk in rural locations also goes hand in hand with socio-economic disadvantage [14], which has been identified as a factor impacting injury risk [15,16,17,18]. Effective injury prevention strategies must consider these (and other) determinants of health when identifying areas of need and designing interventions.

Children and adolescents experience significant fatal and non-fatal burden due to injuries [19] and are particularly vulnerable to harm due to drowning [2,20], falls [3,21] and road traffic injuries [4,22]. Reducing injury-related mortality and morbidity is vital in order for nations to meet child and adolescent health targets within the Sustainable Development Goals [23,24]. Reducing injury-related harm among children and adolescents represents the area where the greatest health gains can be made [25].

While fatal child injury rates are declining, there are clear variations based on socioeconomic inequalities [26]. Therefore, within an Australian context, this study aims to explore injury-related mortality among children and adolescents to identify the impact of determinants of health, specifically rurality and a composite measure of socio-economic advantage and disadvantage (Index of Relative Socio-economic Advantage and Disadvantage (IRSAD)) of residential location, with an aim to inform future prevention efforts.

## 2. Materials and Methods

This study reports a total population analysis of injury-related mortality among children and adolescents aged 0–19 years (henceforth referred to as children) in Australia between 1 January 2007 and 30 June 2017 (a period of 10 years), with a particular focus on determinants of health—namely remoteness and IRSAD of the child’s residential location.

### 2.1. Data Source 

Cause of Death Unit Record File data were sourced from the Australian Bureau of Statistics (ABS). Data are provided to approved applicants only, but similar publicly available data to the Cause of Death data release collated by the ABS annually are provided [27]. Variables made available for analysis were date of death, sex, age group, jurisdiction of death (Australian state or territory and statistical local area), International Classification of Disease (ICD)-10 cause of death code and statistical local area. A statistical local area is a geographical area as used by the ABS. This study specifically uses statistical area level 2 which represents a community that interacts together socially and economically [28].

### 2.2. Case Identification and Data Cleaning

All deaths that had a primary cause of death injury ICD-10-AM [29] code were selected; only cases where the incident occurred during the study period were included, people who were aged less than 20 years were included and those who resided in Australia (i.e., visitors to Australia were excluded). Prior to commencing data coding and analysis, a total of 75 cases were removed; being 54 overseas residents and 21 with unknown residence.

This study examines injury-related deaths that were registered and who died between 1 January 2007 and 31 December 2017; noting that particularly for 2017, this would represent an approximate under-numeration of 6% (this proportion is based on the proportion of people who died within a given year but whose death was not registered until the following year). This particularly impacts those deaths which occur later in the year i.e., November and December. As such, trends over time are explored on Australian financial years 1 July to 30 June, from 1 July 2007 to 30 June 2017.

### 2.3. Coding of Injury Mechanisms

Fifteen categories of injury mechanism were collated using the ICD-10 codes. Due to small numbers of cases, the mechanisms of ‘overexertion, strenuous and repetitive movements’ (X50) and ‘contact with venomous animals and plants’ (X20-29) were grouped into ‘Other’. The coding structure for the categories is described in Table 1.

### 2.4. Coding of Determinants of Health

The impact of determinants of health on child injury risk was explored by remoteness and IRSAD. The remoteness of the child’s residential location was calculated by matching the nine digit statistical local area (SLA) code to the corresponding Australian Standard Geographical Classification (ASGC) category (i.e., major cities, inner regional, outer regional, remote and very remote) [30].

The nine-digit SLA was also used to code the victim’s residential location to the corresponding decile on the index of socio-economic advantage and disadvantage (IRSAD). IRSAD aligns the statistical local area with a decile ranking (1–10), with areas ranked 1 being the most disadvantaged. Victims’ residential postcode current IRSAD was used as a proxy for their familial socio-economic status [31]. IRSAD includes 17 measures around: income, education, employment, car ownership, internet connection, disability, family structure and renting status [32], which are combined to produce 10 IRSAD deciles. The IRSAD deciles were coded to low (deciles 1–3), mid (deciles 4–7) and high (deciles 8–10) for ease of analysis. Data between 2006 and 2011 were coded to the socio-economic indexes for areas (SEIFA) classification in 2011 and data between 2012 and 2017 were coded to SEIFA 2016 [33].

### 2.5. Statistical Analysis

Temporal trends over time in fatal injury mechanism were explored using the linear calculation in Microsoft Excel 365 (Build: 13426.20274). Crude rates and relative risk (RR) with a 95% confidence interval (CI) were used to calculate the impact of remoteness of residential location on injury-related fatalities. Crude rates per 100,000 population were calculated for all children 0–19 years, by sex and by age group (i.e., 0–4 years, 5–9 years, 10–14 years and 15–19 years), using the population from June of each year [34]. Population data by ASGC classification are currently only available for years in which the national census has been conducted. Therefore, a two-year average for the population was calculated using census years (2011 [35] and 2016 [36]) and this was used with a 10-year average of the deaths to calculate crude annualized injury-related fatality rates for children per 100,000 population by ASGC remoteness classification. Rates were used to calculate relative risk (using a MedCalc calculator [37]), with a 95% confidence interval using the lowest rate as the control group.

Univariate and chi-square analyses (calculated in International Business Machines [IBM] Corporation Statistical Package for the Social Sciences [SPSS] V25 [38]) were used to explore the impact of IRSAD on injury-related fatalities. Population data by grouped IRSAD decile (low, mid, and high) and age group are not publicly available in Australia. Therefore, the proportional of the all-age population in Australia as at 2016 by grouped IRSAD decile was calculated and assumed to hold true for children aged 0–19 years across the study period. These proportions were used to calculate non-parametric chi-square tests of significance. A modified Bonferonni correction, as suggested by Keppel [39], was applied at the 0.05 level, where multiple categories within a variable have been analyzed.

### 2.6. Ethics Approval

This study received ethics approval from the James Cook University Human Research Ethics Committee (H6136). Due to ethical constraints associated with reporting small numbers, small cell counts less than five (and their associated percentages) are reported as not presented (NP).

## 3. Results

**Figure 1:**
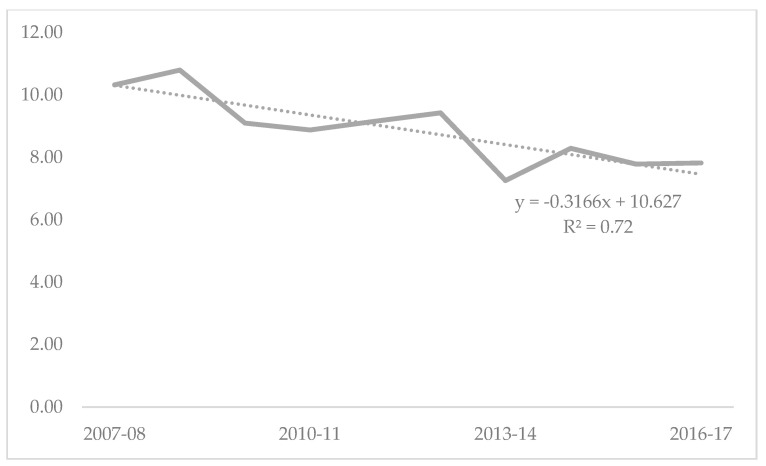
Crude rate per 100,000 of injury-related fatalities among children and adolescents 0–19 years, Australia 2007-08 to 2016-17.

Across the study period, there were a total of 5153 children who died due to fatal injuries. As a rate per 100,000 population, injury-related mortality among 0–19-year-olds in Australia varied from a high of 10.80 in 2008-09 to a low of 7.26 in 2013–14. The temporal trend across the study period shows a decline (y = −0.3166x + 10.627; R^2^ = 0.72) (Figure 1).

**Figure 2:**
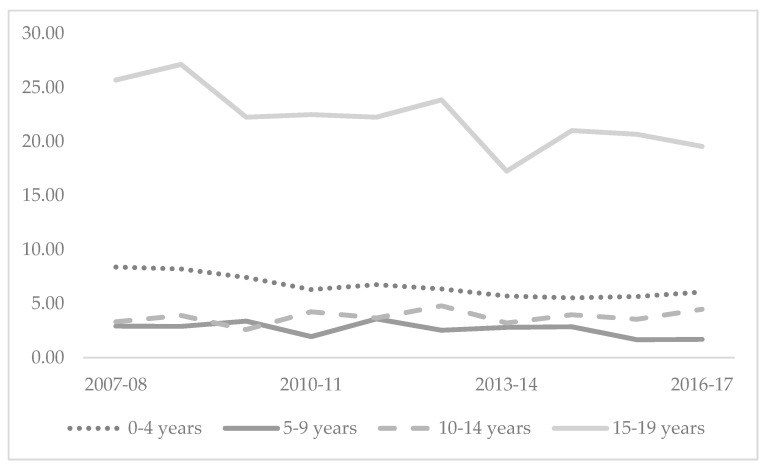
Crude rate per 100,000 of injury-related fatalities among children and adolescents 0–19 years, Australia 2007-08 to 2016-17.

The highest rates of fatal injury were seen in the 15–19 years age group with an average rate across the study period of 22.23 per 100,000, compared with the lowest rate of 2.63 per 100,000 among the 5–9 years age group. Crude injury-fatality rates are declining among 15–19-year-olds, with stagnant rates among 0–4-year-olds and 5–9-year-olds, with a slight upturn in rates among 10–14-year-olds (Figure 2).

Males accounted for 67.7% of all fatalities (*n* = 3487). Males were overrepresented in all age groups, rising from 59.1% of all injury-related fatalities in the 0–4 years age group, to 71.8% of all fatalities in the 15–19 years age group.

Road traffic and other land transport incidents was the leading injury mechanism, accounting for 38.2% (*n* = 1970) of the overall injury-related fatality burden, followed by intentional self-harm (*n* = 1432; 27.8%) and drowning (*n* = 419; 8.1%). The overrepresentation of males was most pronounced in injury-related fatalities as a result of falls (82.6% male) and electrocution, radiation and extreme temperatures (80.0% male). When calculated as crude fatality rates, road traffic and other land transport incidents recorded a fatality rate of 3.39 per 100,000 population, compared to 2.46 for intentional self-harm and 0.72/100,000 population for drowning (Table 2).

### 3.1. Impact of Social Determinants on Injury-Related Fatalities—Remoteness Classification

The rate of injury-related fatalities increased as remoteness increased. Major cities record a crude rate of 6.64 injury-related fatalities per 100,000 residents; rising to a rate of 38.90 per 100,000 residents in very remote areas. Very remote areas recorded injury-related fatalities at six times (RR = 5.84; CI: 3.76–9.12) the rate of major city residents (Table 3).

Males recorded higher rates of fatal injury than females across all remoteness classifications, with the highest rates seen in very remote areas (12.52 for males compared to 9.79 for females). When compared to major cities, the relative risk of an injury-related fatality was six higher in very remote areas for males (RR = 5.62; CI: 3.20–9.87) and nine times higher for females (RR = 8.73; CI: 4.23–18.00). Rates of injury-related fatalities were highest for 15–19-year-olds across all remoteness classification, ranging from 17.11 fatalities per 100,000 residents in major cities, to a rate of 102.33 in very remote areas (Table 4).

Across the five remoteness classification categories, road traffic and other land transport incidents (2.45) and intentional self-harm (2.12) recorded the highest rates of fatal injury in areas classified as major cities. This pattern continued across all remoteness classifications, with the exception of very remote areas, where the rate of injury-related fatalities associated with intentional self-harm (16.97) overtook that of road traffic and other land transport (14.39). The highest RR of injury-related fatality was for electrocution, radiation and extreme temperatures, with an 18 times (RR = 18.24; CI: 0.02–18638.89) higher risk of dying from this injury mechanism in a very remote area than in a major city. (Table 5).

When compared to females, a proportionately higher number of males in the 0–4 years age group died from road transport and other land transport injuries in major cities (57.1%), rising to 79.3% for the 15–19 years age group in very remote areas. When compared to males, a proportionately higher number of females aged 10–14 years died due to road transport related injuries in very remote areas (62.5%). For intentional self-harm related fatalities, sex differences were most pronounced among 15–19-year-olds in inner regional areas, where males accounted for 73.1% of fatalities. Sex differences for drowning were most pronounced in major cities for the 15–19 years age group, with males accounting for 90.9% of all drowning-related fatalities.

### 3.2. Impact of Social Determinants on Injury-Related Fatalities—Socio-Economic Classification

**Figure 3:**
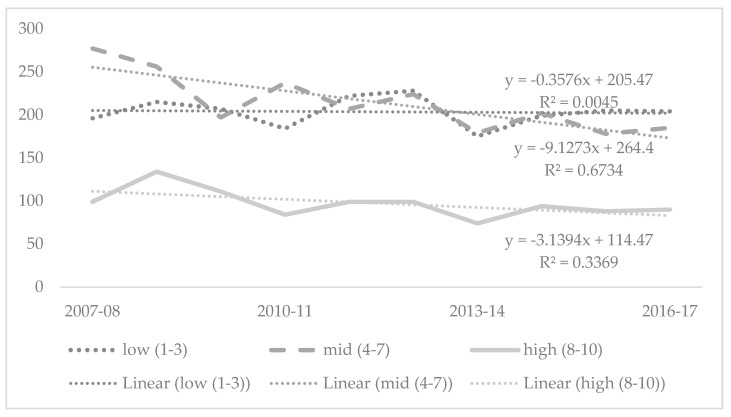
Injury-related fatalities among children and adolescents 0–19 years by index of relative socio-economic advantage and disadvantage (IRSAD) decile, Australia 2007-08 to 2016-17. Note: there were four cases with unknown IRSAD.

There was an annual average of 214 injury-related fatalities in areas classified as mid IRSAD decile, followed by 204 fatalities for low IRSAD decile residences. High IRSAD deciles recorded the lowest average, with 97 injury-related fatalities annually. Injury-related fatalities declined in all three IRSAD decile classifications across the study period, with the largest decrease occurring in the mid decile (y = −9.1273x + 264.4; R^2^ = 0.6734). (Figure 3).

The highest proportion of injury-related fatalities occurred in areas classified as being mid IRSAD deciles (41.6%) and among males (67.7%). Males accounted for a higher proportion of injury-related fatalities than females across all IRSAD deciles. A higher proportion of female injury-related fatalities occurred among high IRSAD deciles (20.0%) than males (18.4%), however sex by IRSAD decile did not have a statistically significant impact on injury-related fatalities (X^2^ = 1.86; *p* = 0.395). (Table 6)

Across all age groups, the highest proportion of injury-related fatalities occurred in areas classified as low IRSAD, with the exception of the 15–19 years age group, where the highest proportion occurred in areas classified as mid IRSAD (36.9%). There was a statistically significant difference in injury-related fatalities for age group by IRSAD. Older children (15–19-year-olds) were more likely to die from injury-related incidents in areas classified as high IRSAD, whereas children (0–4 years) were more likely to die from injury-related incidents if residing in low IRSAD areas (X^2^ = 28.58; *p* < 0.001) (Table 7).

Road traffic and other land transport was the leading mechanism of injury-related fatalities in areas classified as low IRSAD (38.9%) and mid IRSAD (40.7%). In areas classified as high IRSAD, intentional self-harm accounted for the highest proportion of injury-related deaths (32.4%). Statistically significant differences (X^2^ = 69.05; *p* < 0.001) were found for proportion of injury mechanism by IRSAD for road traffic and other land transport (more likely in low and mid IRSAD deciles), accidental poisoning (high IRSAD), and intentional self-harm (low and mid IRSAD) (Table 8).

When exploring sex differences by injury mechanism, age and IRSAD decile, 15–19-year-old males accounted for a significantly higher proportion of drowning-related deaths in low (96.3% male), mid (89.2%) and high (90.9%) IRSAD deciles. Females aged 0–4 years old accounted for 71.4% of burn-related deaths in high IRSAD deciles. A higher proportion of males aged 15–19 years of age died from intentional self-harm in low IRSAD deciles (72.7% male) compared to mid (68.5% male) and high IRSAD deciles (66.1% male).

## 4. Discussion

Injury is a leading, yet preventable cause of death among children. This study has explored the impact of determinants of health, namely remoteness of residential location and IRSAD of residential location on injury-related fatalities among 0–19-year-olds in Australia. This study reports the injury-related fatalities of 5153 children aged 0–19 years, with the highest rates occurring among adolescents 15–19 years (22.23/100,000 population). The highest rates of injury-related fatalities being due to the mechanisms of road traffic and other land transport (3.39), intentional self-harm (2.46) and drowning (0.72). Rates of injury-related fatality increased as remoteness increased, with six times the risk of an injury-related fatality for children aged 0–19 years in very remote areas of Australia when compared to those residing in major cities. Males and 15–19-year-olds recorded the highest rates of injury-related fatality across all remoteness classifications and age bands, with the highest rate seen among 15–19-year-olds in very remote areas (a rate of 102.33/100,000 people).

Variations in fatal injury risk also exist based on the socio-economic advantage or disadvantage of where children live. While sex did not significantly impact injury-related fatalities when examined by IRSAD decile, significant differences were seen by age band and injury mechanism. Fatal injury risk was most pronounced in low IRSAD areas for very young children (0–4 years of age) and in high IRSAD areas for 15–19-year-olds. Road transport and other land transport injury-related fatalities and intentional self-harm fatalities were significantly more likely in low and mid IRSAD deciles, while accidental poisoning-related fatalities were significantly more likely in high IRSAD deciles. We now focus our discussion on the three leading injury mechanisms identified in the study being road traffic and other land-based transport, intentional self-harm and drowning. We then look to the future with respect to preventative efforts, while also focusing on the opportunity that the new National Injury Prevention Plan provides.

Road traffic and other land-based transport incidents accounted for the largest number of fatalities (*n* = 1970, 38%) across the study period. High rates were seen across all remoteness classifications, with the fatality risk associated with this mechanism six times higher (RR = 5.87; CI: 2.83–12.18) in very remote areas when compared to major cities. Similarly, road traffic injuries were significantly more likely in areas classified as low (n = 791, 40%) and mid (*n* = 871, 42%) IRSAD deciles. Road surfaces are generally of poorer quality in regional and remote Australia [40], there is a greater diversity of vehicles on the road [41], lower populations see lower investment in vital transport-related infrastructure [42] and higher speeds and driver fatigue all contribute to high fatality rates on regional Australian roads [43]. This, combined with regional dwelling populations often having to travel longer distances by road to seek major services including medical care [44,45,46], leads to increased exposure and risk of injury and death. Investment in higher quality rural roads which allow for passing and accommodate the wider range of vehicles may be a strategy to reduce injury-related fatalities [43], while upstream, policy approaches, such as investing in more regionally based doctors and specialist services [46] may also be required.

Socio-economic status impacts the mode of transport used [47]. For residents of low-socio economic areas, the use of lower cost, older vehicles with poorer safety standards may be contributing to an increased risk of death [43]. Strategies to combat this may be policy approaches such as subsidies to improve vehicle quality and investment in improving infant restraint fitting and correct use [48]. Research also indicates that residents of low socio-economic areas have higher exposure to traffic [49] and see lower investment in transport-related infrastructure [50]. Similar to addressing road traffic-related mortality in geographically isolated areas, multi-faceted strategies will be needed to reduce injury risk. For children and adolescents who are too young to drive, such strategies must take a whole community level approach and or target parents and caregivers. 

Intentional self-harm was the second leading cause of injury-related mortality among children in Australia across the study period, accounting for 28% of all deaths. Intentional self-harm recorded the highest fatality rate of any mechanism in very remote areas (16.97 per 100,000 residents), a rate that is eight times (RR = 8.02; CI: 4.06–15.84) that of the fatality rate in major cities. Adolescence is a period of high risk for suicide, due to multiple stressors, mood disorders and development phase [51,52]. Though legislation in Australia significantly limits firearm availability and familiarity among potential users, compared to firearm-heavy nations such as the United States [53], increased access to firearms in rural areas may be a contributing factor to increased risk of intentional self-harm-related death in remote areas of Australia [54,55].

Like most other injury mechanisms explored, this study identified that the risk of an intentional self-harm-related fatality was significantly higher for residents of low and mid IRSAD deciles. The published literature has also identified this increased intentional self-harm risk in areas of socio-economic disadvantage [56,57]. Given the impact of broader social factors on risk of intentional self-harm, upstream social and economic approaches that seek to redress socio-economic disadvantage of children and adolescents in Australia are recommended, beyond the provision of mental health services [58]. 

This study found drowning to be the third leading cause of injury-related fatalities among children in Australia. Like other injury mechanisms, rates increased as remoteness increased, aside from a slightly higher rate in inner regional (1.45 per 100,000 residents) than outer regional (1.41), with a four times (RR = 4.05; CI: 0.74–22.22) greater risk of drowning in very remote areas than in major cities. Similarly, a higher proportion of drowning occur in areas of low and mid socio-economic status, with just 17.2% of drowning deaths occurring in high socio-economic areas. This is in keeping with other studies into drowning which have identified significantly higher rates of drowning as rurality increases [59,60] and in low socio-economic areas [61,62].

A range of upstream factors also impact drowning risk, with less pools, seasonal facilities and a lack of instructors impacting ability to learn to swim in regional and remote areas [59], and the limited provision of school-based lessons and the high cost of private lessons a barrier for low-socio economic families [63]. Unpatrolled inland waterways used for recreation [60] and higher rates of alcohol consumption combined with aquatic activity [64], are also factors increasing drowning risk in regional and remote areas. Social determinants, such as remoteness and socio-economic disadvantage, are important factors impacting drowning risk and must be considered by drowning prevention practitioners. Very few drowning prevention initiatives targeting regional and remote communities have been evaluated [59], providing meagre evidence to direct limited funding to the most effective strategies.

Greater investment is clearly needed in primary prevention measures to reduce injury-related mortality and morbidity in regional and remote Australia and among areas of socio-economic disadvantage [65]. This will require also addressing the determinants of health and taking a wider lens when developing prevention programs, including ensuring programs are piloted in rural areas, engaging with families through co-design [66] and investing in infrastructure in low socio-economic areas [67]. Primary prevention measures must however, go hand in hand with improved tertiary prevention strategies, such as community first aid and cardiopulmonary resuscitation (CPR) [68] and trauma care services [69,70].

The existence of the new National Injury Prevention Strategy 2020–2030 [71], which identifies the impact of determinants of health such as remoteness and socio-economic status in many of its priority populations and priority areas, may serve as a driver to generate policy change and increased investment in addressing groups at increased injury risk due to determinants of health. 

Preventing injury-related harm is vital to the nation’s addressing many of the child and adolescent health targets outlined in the Sustainable Development Goals [23,24]. More broadly, injury prevention efforts intersect many global agendas such as healthy aging, climate change, water safeguarding, urbanization and corporate social responsibility [24]. As part of the United Nations’ Decade of Action to 2030, leveraging the broader impacts of, and intersections with, injury prevention may present opportunities for greater political commitment to tackling this preventable cause of mortality and morbidity. This study has identified opportunities for further research, in particular the need for greater implementation and evaluation of injury prevention interventions targeted at residents of regional and remote and low socio-economic areas of Australia across a range of injury mechanisms. Advocacy to encourage greater investment in injury prevention in these disadvantaged areas will be strengthened by the evidence to support the best return on investment. 

### Strengths and Limitations 

This study adds to the limited literature exploring the impact of injury related fatality among children aged 0–19 years in Australia by determinants of health, namely remoteness and socio-economic status of residential location at a total population level. The study uses a nationally validated data source and is likely to have consistency and accuracy with respect to the identification of cause of death. Where population data were available, this study uses rates to explore risk of injury-related fatalities per 100,000 residents.

There are, however, some limitations associated with this study. This study explores fatalities only, and further research is needed to explore the full burden of injury-related morbidity. Population data by IRSAD decile are not publicly available in Australia and therefore we were unable to calculate fatality rates by IRSAD. Assumption made regarding population distribution across the all-age population grouped IRSAD deciles holding true for children aged 0–19 years may not be correct. As such, the chi square tests of significance should be interpreted with caution. As remoteness and IRSAD are calculated at the statistical local area level, this limits any individual advantage or disadvantage which may occur within a statistical local area. For example, in an area considered to be low IRSAD, it is possible that there are households which would be considered high within the IRSAD classification. With respect to remoteness, some of the statistical areas are quite large, potentially spanning several remoteness classifications, however in this study, they have been assigned to a single remoteness classification. 

## 5. Conclusions

This study has explored the impact of determinants of health, namely the geographical remoteness of residence and socio-economic status of residential area on injury-related fatalities among children aged 0–19 years in Australia. This study identified road traffic and other land transport injuries, intentional self-harm and drowning to be the three leading causes of death, with fatality rates increasing as remoteness increased. For most injury mechanisms examined that there was a disproportionate burden among low and middle IRSAD deciles except for intentional self-harm and accidental poisoning related deaths, which were significantly higher in high IRSAD areas. The findings of this study suggest injury prevention strategies for young people in Australia should consider these determinants of health. People residing in regional and remote areas and from low socio-economic backgrounds already face significant health and lifestyle challenges associated with disadvantage. It is time to invest in these populations to minimize any further preventable loss of life. 

## Figures and Tables

**Table 1:** Injury categories used in study and associated International Classification of Diseases (ICD)-10 codes.

Injury Mechanisms Category.	ICD-10 Code	Code Explanation
Road traffic and other land transport	V00–V09	Pedestrian injured in transport accident
	V10–V19	Pedal cycle rider injured in transport accident
	V20–V29	Motorcycle rider injured in transport accident
	V30–V39	Occupant of three-wheeled motor vehicle injured in transport accident
	V40–V49	Car occupant injured in transport accident
	V50–V59	Occupant of pick-up truck or van injured in transport accident
	V60–V69	Occupant of heavy transport vehicle injured in transport accident
	V70–79	Bus occupant injured in transport accident
	V80–89	Other land transport accidents
Water transport, air and space transport and other/unspecified	V90–94	Water transport accidents
	V95–V97	Air and space transport accidents
	V98–V99	Other and unspecified transport accidents
Falls	W00–W19	Slipping, tripping, stumbling and falls
Exposure to mechanical forces	W20–W49	Exposure to inanimate mechanical forces
	W50–W64	Exposure to animate mechanical forces
Drowning	W65–74	Accidental non-transport drowning and submersion
Other accidental threats to breathing	W75–W84	Other accidental threats to breathing
Electrocution, radiation and extreme temperature	W85–W99	Exposure to electrical current, radiation and extreme ambient air temperature and pressure
Burns	X00–X09	Exposure to smoke, fire and flames
	X10–X19	Contact with heat and hot substances
Forces of nature	X30–X39	Exposure to forces of nature
Accidental poisoning	X40–X49	Accidental poisoning by and exposure to noxious substances
Accidental exposure to other forces	X51–X59	Accidental exposure to other specified factors
Intentional self-harm	X60–X84	Intentional self-harm
Assault	X85–Y09	Assault
Undetermined intent	Y10–Y34	Event of undetermined intent
Other	X20–X29	Contact with venomous animals and plants
	X50	Overexertion and strenuous or repetitive movements
	Y35	Legal intervention and operations of war
	Y40–Y84	Complications of medical and surgical care
	Y85–Y89	Sequelae of external causes of morbidity and mortality

**Table 2:** Incidence of injury-related fatality by mechanism, proportion of total and crude rate per 100,000 population among children and adolescents 0–19 years of age, Australia, 2007-08–2016-17.

Injury Mechanism	Incidence (*n* =)	Proportion of Total (%)	Crude Rate/100,000 Population
Total	5153	100.0	8.86
Road traffic and other land transport	1970	38.2	3.39
Water transport, air and space transport and other/unspecified	39	0.8	0.07
Falls	92	1.8	0.16
Exposure to mechanical forces	125	2.4	0.21
Drowning	419	8.1	0.72
Electrocution, radiation and extreme temperatures	15	0.3	0.03
Burns	112	2.2	0.19
Forces of nature	35	0.7	0.06
Accidental poisoning	167	3.2	0.29
Accidental exposure to other forces	39	0.8	0.07
Intentional self-harm	1432	27.8	2.46
Assault	292	5.7	0.50
Undetermined intent	144	2.8	0.25
Other accidental threats to breathing	228	4.4	0.39
Other	44	0.9	0.08

**Table 3:** Crude rate of injury-related fatality by remoteness classification, relative risk with 95% confidence interval, children and adolescents 0–19 years of age, Australia, 2007-08–2016-17.

Remoteness Classification	Proportional Population Distribution (%)	Crude Rate of Injury-Related Fatality/100,000 Population	Relative Risk (95% Confidence Interval)
Major Cities	69.9	6.64	1
Inner Regional	18.8	12.48	1.88 (1.52–2.31)
Outer Regional	9.0	15.41	2.32 (1.80–2.98)
Remote	1.4	25.75	3.88 (2.46–6.10)
Very Remote	1.0	38.90	5.86 (3.76–9.12)

**Table 4:** Crude rate of injury-related fatality by remoteness classification, relative risk (RR) with 95% confidence interval (CI), children and adolescents 0–19 years of age, Australia, 2007-08–2016-17.

Crude Rate of Injury-Related Fatality/100,000 Population										
	Major Cities	RR (95% CI)	Inner Regional	RR (95% CI)	Outer Regional	RR (95% CI)	Remote	RR (95% CI)	Very Remote	RR (95% CI)
Sex										
Male	2.27	1	4.53	2.00 (1.55–2.57)	5.37	2.39 (1.76–3.24)	9.16	4.04 (2.35–6.97)	12.52	5.62 (3.20–9.87)
Female	1.08	1	1.93	1.77 (1.22–2.57)	2.58	2.36 (1.51–3.68)	4.40	4.00 (1.75–9.16)	9.79	8.73 (4.23–18.00)
Age Group										
0–4 years	4.94	1	8.80	1.78 (1.08–2.93)	14.65	2.97 (1.74–5.06)	18.23	3.69 (1.33–10.21)	24.43	4.94 (1.72–14.20)
5–9 years	1.63	1	4.11	2.53 (1.17–5.44)	5.11	3.15 (1.28–7.74)	9.95	6.12 (1.45–25.77)	12.49	7.68 (1.71–34.55)
10–14 years	2.74	1	4.67	1.70 (0.87–3.33)	5.76	2.10 (0.94–4.71)	2.48	0.90 (0.26–3.17)	32.17	11.73 (4.14–33.26)
15–19 years	17.11	1	32.31	1.89 (1.46–2.45)	37.48	2.19 (1.58–3.03)	71.63	4.18 (2.29–7.64)	102.33	5.97 (3.28–10.88)

**Table 5:** Crude rate per 100,000 population of injury-related fatality by mechanism and remoteness classification, relative risk (RR) and 95% confidence interval (CI), among children and adolescents 0–19 years of age, Australia, 2007-08–2016-17.

Injury Mechanism	Crude Rate of Injury-Related Fatality/100,000 Population									
	Major Cities	RR (95% CI)	Inner Regional	RR (95% CI)	Outer Regional	RR (95% CI)	Remote	RR (95% CI)	Very Remote	RR (95% CI)
Road traffic and other land transport	2.45	1	6.24	2.55 (1.86–3.48)	7.37	3.01 (2.06–4.38)	10.77	4.39 (2.17–8.89)	14.39	5.87 (2.83–12.18)
Water transport, air and space transport and other/unspecified	0.06	1	0.08	1.34 (0.12–14.92)	0.16	2.47 (0.20–30.66)	0.26	4.05 (0.04–385.50)	0.18	2.92 (0.01–1622.28)
Falls	0.15	1	0.18	1.20 (0.23–6.15)	0.25	1.70 (0.25–11.37)	0.64	4.30 (0.24–77.05)	0.37	2.47 (0.03–213.07)
Exposure to mechanical forces	0.14	1	0.35	2.46 (0.66–9.15)	0.49	3.45 (0.78–15.32)	1.15	8.15 (0.88–75.44)	2.03	14.33 (1.86–110.64)
Drowning	0.64	1	1.05	1.65 (0.82–3.35)	1.45	2.27 (1.00–5.15)	1.41	2.21 (0.33–14.93)	2.58	4.05 (0.74–22.22)
Electrocution, radiation and extreme temperatures	0.01	1	0.06	5.59 (0.10–305.20)	0.04	3.86 (0.02–828.20)	0.26	25.34 (0.12–5432.68)	0.18	18.24 (0.02–18638.89)
Burns	0.14	1	0.40	2.79 (0.79–9.90)	0.25	1.79 (0.27–12.09)	0.26	1.81 (0.02–156.60)	0.92	6.51 (0.36–117.53)
Forces of nature	0.05	1	0.10	1.95 (0.19–19.59)	0.10	3.68 (0.34–39.81)	0.13	2.41 (0.00–1373.41)	0.18	3.47 (0.01–1976.44)
Accidental poisoning	0.31	1	0.37	1.20 (0.38–3.76)	0.20	0.64 (0.08–4.91)	0.64	2.09 (0.12–35.44)	0.92	3.01 (0.18–51.00)
Accidental exposure to other forces	0.08	1	0.08	1.08 (0.10–11.30)	0.06	0.75 (0.02–31.72)	0.13	1.64 (0.00–887.94)	0.37	4.71 (0.05–432.97)
Intentional self-harm	2.12	1	3.22	1.52 (1.02–2.26)	4.32	2.04 (1.28–3.26)	7.69	3.63 (1.59–8.32)	16.97	8.02 (4.06–15.84)
Assault	0.50	1	0.41	0.83 (0.30–2.34)	0.96	1.92 (0.71–5.17)	1.03	2.06 (0.22–19.25)	1.11	2.22 (0.17–28.99)
Undetermined intent	0.20	1	0.51	2.58 (0.86–7.72)	0.43	2.18 (0.49–9.73)	0.38	1.95 (0.05–74.75)	0.55	2.81 (0.07–107.57)
Other accidental threats to breathing	0.34	1	0.47	1.40 (0.50–3.91)	0.72	2.15 (0.68–6.80)	1.92	5.72 (1.06–30.92)	1.29	3.84 (0.35–42.47)
Other	0.07	1	0.10	1.41 (0.71–2.83)	0.18	2.40 (1.14–5.07)	0.26	3.50(0.83–14.65)	0.00	UTBC

UTBC = Unable to Be Calculated.

**Table 6:** Injury-related fatalities by sex and index of relative socio-economic advantage and disadvantage decile (IRSAD), Australia, 2007-08 to 2016-17.

Sex	IRSAD Decile								X^2^ (*p* Value)
	Total		Low (Deciles 1–3)		Mid (Deciles 4–7)		High (Deciles 8–10)		
	*N*	%	*N*	%	*N*	%	*N*	%	
Total	5149	100.0	2035	39.5	2142	41.6	972	18.9	
Male	3485	67.7	1387	39.8	1458	41.8	640	18.4	1.857 (*p* = 0.395)
Female	1664	32.3	648	38.9	684	41.1	332	20.0	

Note: excludes four cases with unknown IRSAD classification.

**Table 7:** Injury-related fatalities by age group and index of relative socio-economic advantage and disadvantage decile (IRSAD), Australia, 2007/08 to 2016/17.

Age Group of Injury-Related Fatalities	IRSAD Decile								X^2^ (*p* Value)
	Total		Low (Deciles 1–3)		Mid (Deciles 4–7)		High (Deciles 8–10)		
	*N*	%	*N*	%	*N*	%	*N*	%	
Total	5149	100.0	2035	39.5	2142	41.6	972	18.9	
0–4-year-olds	988	19.2	431 _a_	43.6	398 _a,b_	40.3	159 _b_	16.1	28.579 (*p* < 0.001)
5–9-year-olds	378	7.3	165 _a_	43.7	144 _a_	38.1	69 _a_	18.3	
10–14-year-olds	531	10.3	240 _a_	45.2	200 _b_	37.7	91 _a,b_	17.1	
15–19-year-olds	3252	63.2	1199 _a_	36.9	1400 _b_	43.1	653 _b_	20.1	

Note: excludes four cases with unknown IRSAD classification. Each subscript letter (_a,b_) denotes a subset of IRSAD Grouped into Low Mid High categories whose column proportions do not differ significantly from each other at the 0.05 level using the Bonferroni adjustment (i.e., where there are two a’s these are not statistically significant, where there is an a and b these are statistically significant).

**Table 8:** Injury-related fatalities by age group and index of relative socio-economic advantage and disadvantage decile (IRSAD), Australia, 2007-08 to 2016-17.

Mechanism of Injury-Related Fatalities	IRSAD Decile								X^2^ (*p* Value)
	Total		Low (Deciles 1–3)		Mid (Deciles 4–7)		High (Deciles 8–10)		
	*N*	%	*N*	%	*N*	%	*N*	%	
Total	5149	100.0	2035	39.5	2142	41.6	972	18.9	-
Road traffic and other land transport	1970	38.3	791 _a_	40.2	871 _a_	44.2	308 _b_	15.6	69.052 (*p* < 0.001)
Water transport, air and space transport and other/unspecified	39	0.8	8 _a_	20.5	24 _b_	61.5	7 _a,b_	17.9	
Falls	92	1.8	29 _a_	31.5	40 _a_	43.5	23 _a_	25.0	
Exposure to mechanical forces	125	2.4	47 _a_	37.6	56 _a_	44.8	22 _a_	17.6	
Drowning	418	8.1	175 _a_	41.9	171 _a_	40.9	72 _a_	17.2	
Electrocution, radiation and extreme temperatures	15	0.3	5 _a_	33.3	6 _a_	40.0	NP _a_	NP	
Burns	111	2.2	38 _a_	34.2	55 _a_	49.5	18 _a_	16.2	
Forces of nature	35	0.7	12 _a_	34.3	16 _a_	45.7	7 _a_	20.0	
Accidental poisoning	167	3.2	52 _a_	31.1	72 _a,b_	43.1	43 _b_	25.7	
Accidental exposure to other forces	39	0.8	12 _a_	30.8	12 _a_	30.8	15 _b_	38.5	
Intentional self-harm	1431	27.8	555 _a_	38.8	561 _a_	39.2	315 _b_	22.0	
Assault	292	5.7	124 _a_	42.5	110 _a_	37.7	58 _a_	19.9	
Undetermined intent	143	2.8	65 _a_	45.5	54 _a_	37.8	24 _a_	16.8	
Other accidental threats to breathing	228	4.4	100 _a_	43.9	80 _a_	35.1	48 _a_	21.1	
Other	44	0.9	22 _a_	50.0	14 _a_	31.8	8 _a_	18.2	

Note: excludes four cases with unknown IRSAD classification. NP = Not Presented. Each subscript letter (_a,b_) denotes a subset of IRSAD Grouped into Low Mid High categories whose column proportions do not differ significantly from each other at the 0.05 level using the Bonferroni adjustment (i.e., where there are two a’s these are not statistically significant, where there is an a and b these are statistically significant).

## Data Availability

Agreements in place for use of data in this study place restrictions on the public storage of such data. Those interested in gaining access to the data used in this study, should contact the Australian Bureau of Statistics via email: client.services@abs.gov.au.
